# A rare case of a migrating fishbone lodged in the larynx for 6 months in a patient with delayed presentation due to COVID-19 pandemic

**DOI:** 10.1093/jscr/rjab131

**Published:** 2021-05-17

**Authors:** Mohamed Alreefi, Noora Althawadi, Ankit Patel, Raghav Dwivedi

**Affiliations:** Head and Neck Surgery Department, University College London Hospital, London, UK; Head and Neck Surgery Department, University College London Hospital, London, UK; Head and Neck Surgery Department, University College London Hospital, London, UK; Head and Neck Surgery Department, University College London Hospital, London, UK

## Abstract

A foreign body sensation following fishbone ingestion is a common presentation to the Accident and Emergency Department. Simple removal is achieved with acute presentations and accessible locations. The risk of complications increases with delay in seeking medical attention. We report a case of a migratory fishbone lodged in the larynx for 6 months in a patient with delayed presentation due to COVID-19 pandemic

## INTRODUCTION

A foreign body sensation following ingestion is a common presentation to the Accident and Emergency department and to otolaryngologists. Fishbones are amongst the most commonly ingested foreign bodies leading to this symptomatic presentation across various age groups. The more common sites of impaction include the oropharynx and the oesophagus [1]. Patients usually present within the first 24–48 hours of ingestion with a foreign body sensation, localized pain or difficulty in swallowing [2]. Delayed presentation may consequentially lead to more life-threatening complications such as perforation, cervical abscess and mediastinitis [5].

We report a case of a large migratory fishbone in a patient who presented 6 months following ingestion, due to the COVID-19 pandemic.

## CASE REPORT

A 51-year-old female presented to the Head and Neck Cancer clinic complaining of right sided throat and neck discomfort for 6 months following fishbone foreign body ingestion. At the time, she did not seek medical attention due to the concerns surrounding the COVID-19 pandemic. Her symptomatic discomfort worsened the few weeks leading to her clinic appointment, but she did not have any other symptoms to report. No red flag symptoms such as odynophagia, interscapular pain, neck lumps, hemoptysis, voice change or weight loss were reported. She had a past medical history of diabetes mellitus, hypertension and hypothyroidism. Oral examination was unremarkable. Neck examination revealed no palpable neck lumps or lymphadenopathy. She did, however, complain of localized tenderness around the right thyroid cartilage region on deep palpation. Fibreoptic nasal endoscopy (FNE) showed a smooth 2 × 1 cm nodule involving the right arytenoid and aryepiglottic fold. The swelling was clinically benign and did not show signs of inflammation. No obvious foreign body or fishbone was seen, and the postnasal space, tongue base, larynx and hypopharynx were unremarkable.

**Figure 1:**
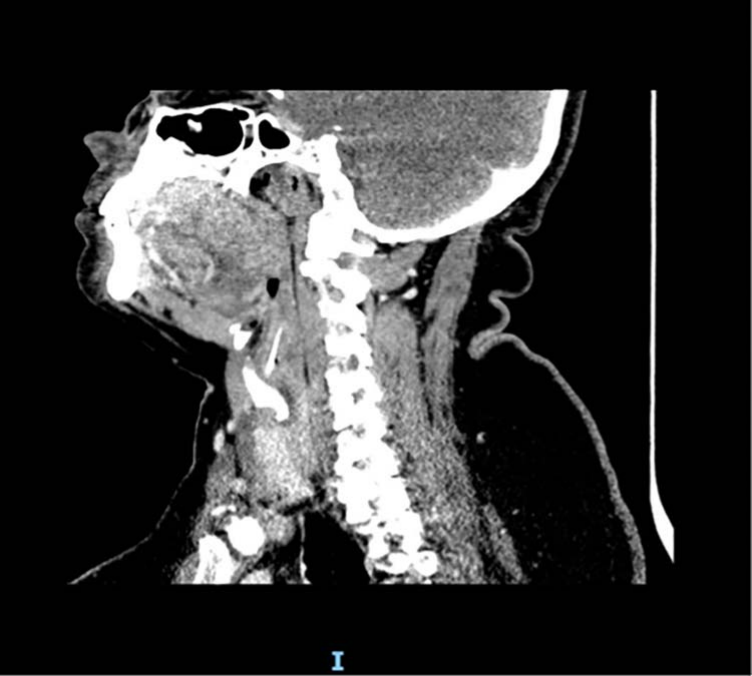
CT scan showing location of fish bone.

**Figure 2:**
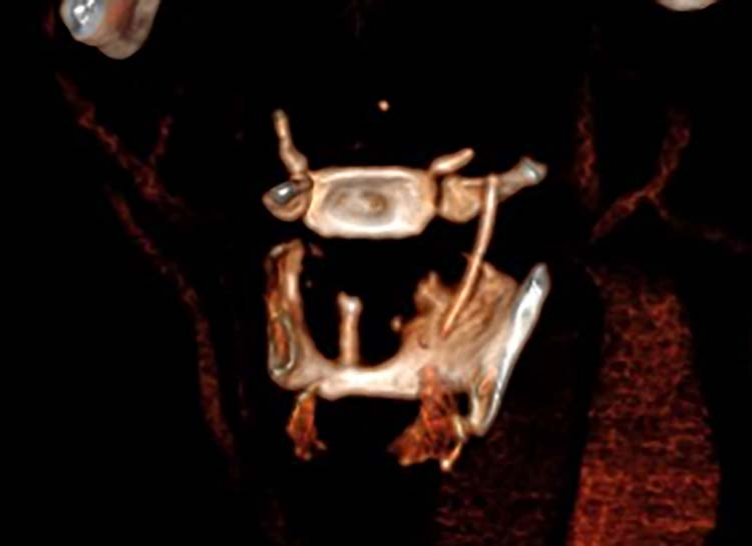
3D reconstruction.

Due to the suspicion of trauma from fishbone ingestion leading to localized swelling and inflammation, a non-contrast computed tomography (CT) scan of the neck was arranged. This revealed a 2.7 cm curvilinear calcific density in the right supraglottic larynx, extending cranially from the anterior wall of the right pyriform fossa, involving the paraglottic fat caudally to the level of the superior aspect of the right vocal cord, 1–2 mm deep to the thyroid lamina and 4–5 mm lateral to the superior surface of the vocal process of the right arytenoid (Figs 1 and 2). The patient was listed for panendoscopy and removal of foreign body.

**Figure 3:**
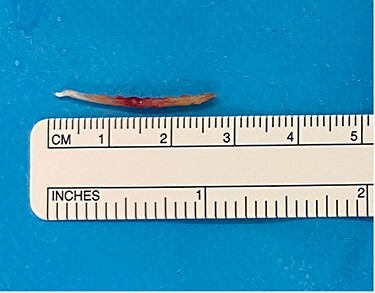
Fishbone after surgical retrieval.

Intraoperatively, a Lindholm scope fixed into suspension and blunt grasping forceps were used to identify a 2.5 cm fishbone posterior to the aryepiglottic fold, extending into the lateral pharyngeal wall (Fig. 3). The fishbone was removed without bleeding or damage to the surrounding structures. The remaining pharynx and oral cavity were unremarkable.

Post-operatively the patient was kept nil by mouth for 2 hours and was slowly built back to a regular diet. She was followed up in the clinic 2 months post-operatively, and her initial symptoms have fully resolved.

## DISCUSSION

Acute presentation following fishbone foreign body ingestion allows for prompt identification and removal of the foreign body to prevent progression from localized symptoms to systemic symptoms and complications. A thorough history and examination, aided by imaging modalities such as lateral plain film radiography of the neck, and detailed CT scanning, all help in diagnosing fishbone foreign body impaction and reduce the risk of complications. These complications include, but are not limited to, migration into surrounding soft tissue, resulting in perforation of key structures, large blood vessel rupture, localized infection and abscess formation, as well as dislodgement into the lower airway [1, 3, 5, 6, 8]. The risk of systemic complications increases with delayed presentation.

In 2013, V Vallamkondu *et al.* reported a case of a neck abscess, 2-weeks following fishbone ingestion in a 57-year-old male with delayed presentation [4]. In the case we have described above, the patient presented 6 months following the initial onset of symptoms and these fortunately did not progress to a more life-threatening situation. The patient described by Vallamkondu presented with anterior neck swelling, thus a neck ultrasound had been organized. The usual modalities of imaging in a suspected case of fishbone foreign body impaction are lateral neck radiography and CT scanning, with the latter being more superior in terms of sensitivity and specificity. CT also allows for identification of complications if present. [6]

The sites of impaction of a fishbone foreign body in the oral cavity and hypopharynx have been described by Kim *et al*. starting with most-frequent: palatine tonsils, base of tongue, vallecula and pyriform sinus [7]. The CT scan for our patient showed that her fishbone was impacted in the pyriform fossa. It is highly likely that if our patient presented earlier, retrieval of the fishbone could have been more straightforward.

The current COVID-19 pandemic has resulted in delayed presentations for many medical and surgical conditions due to the fear of contracting the virus. Certain conditions, maybe perceived by patients to be simple and of low risk, preventing them from seeking medical attention. However, the risks of developing complications increase with delayed presentation; it is important to increase awareness to the public to the importance of early attendance, to reduce morbidity and mortality to the patient, as well as overall healthcare costs. The resultant delayed presentations also require clinicians to be vigilant of this possibility whilst assessing patients in the emergency or outpatient setting.
